# Health and Functional Outcomes for Shared and Unique Variances of Interpersonal Callousness and Low Prosocial Behavior

**DOI:** 10.1007/s10862-019-09756-9

**Published:** 2019-09-15

**Authors:** Alan J. Meehan, Barbara Maughan, Edward D. Barker

**Affiliations:** 1 Social, Genetic & Developmental Psychiatry Centre, Institute of Psychiatry, Psychology & Neuroscience, King’s College London, 16 De Crespigny Park, London SE5 8AF, UK; 2 Department of Psychology, Institute of Psychiatry, Psychology & Neuroscience, King’s College London, 16 De Crespigny Park, London SE5 8AF, UK

**Keywords:** Interpersonal callousness, Low prosocial behavior, Child psychopathy, Psychopathology, Avon Longitudinal Study of Parents and Children (ALSPAC)

## Abstract

Previous factor-analytic studies identify significant comorbidity between interpersonal-callous (IC) traits and low prosocial behavior (LPB), which, in turn, is associated with high levels of childhood risk exposure and psychopathology. Longitudinal associations between IC, LPB, or their combination, and early-adult health and social functioning have not been investigated, however. Extending a previously-identified bifactor model within a prospective birth cohort, this study applied latent path analysis to test direct and indirect pathways (via adolescent delinquency, substance use, and physical activity) between these general and specific factors (age 13) and (i) emotional problems (age 18), (ii) physical health problems (age 18), and (iii) classification as ‘not in education, employment, or training’ (NEET; age 20). All models controlled for childhood adversity and IQ. Bifactor-specific estimates indicated that the residual IC factor did not reliably denote unique variance over and above a general factor (IC/LPB). IC/LPB itself was directly associated with NEET classification, while the residual LPB factor was associated with *better* emotional and physical health. IC/LPB also indirectly associated with emotional problems via greater adolescent delinquency, and with physical health problems via lower physical activity. In contrast, residual LPB variance was either non-significantly or negatively related to these adolescent domains. Findings indicate that the shared variance underlying IC and LPB confers an increased risk for poor health and functional outcomes in emerging adulthood, and highlight delinquency and physical inactivity as potential adolescent treatment targets that may mitigate the risk for health difficulties at high levels of this IC/LPB construct.

The presence of psychopathic traits in childhood and adolescence, particularly its interpersonal (i.e., grandiose, manipulative) and affective features (i.e., low empathy/guilt) consistently characterizes youth with more severe, chronic, and diverse conduct problems (CP; for review, see Frick et al. 2014). This ‘interpersonal-affective’ dimension, commonly referred to as ‘interpersonal callousness’ (IC) in the child and adolescent literature (e.g., Byrd et al. 2012; Byrd et al. 2018; Pardini et al. 2006), aligns with Factor 1 of the Psychopathy Checklist (PCL; Harpur et al. 1989), the prototypical measure of the construct. Of particular note, longitudinal studies have linked IC with poor early-adult developmental outcomes, including delinquency and antisocial personality features (Forsman et al. 2010; McMahon et al. 2010; Pardini and Loeber 2008), criminal arrests and charges (Kahn et al. 2013; Lynam et al. 2009), and both violent and general recidivism (Salekin 2008), even after controlling for a range of delinquency-related risk factors. Indeed, the utility of these traits in distinguishing a high-risk subgroup of young people is reflected in the *DSM-5*’s ‘limited prosocial emotions’ specifier for conduct disorder diagnosis (American Psychiatric Association 2013).

Elsewhere, childhood studies consistently report a moderate association between higher levels of psychopathic traits and lower levels of prosocial behavior (e.g., caring, comforting, or volunteering behaviors; see Eisenberg et al. 2006), with these youth characterized by higher levels of early risk exposure and co-occurring externalizing and internalizing problems (Barker et al. 2011; Meehan et al. 2017). In an exploratory factor analysis of childhood behavioral measures, Dadds et al. (2005) identified a single factor comprised of ‘Callous-Unemotional’ (i.e., PCL Factor 1) items from the Antisocial Process Screening Device (APSD; Frick and Hare 2001; e.g., ‘no guilt’, ‘does not show feelings or emotions’, ‘breaks promises’) and reverse-coded ‘prosocial’ items from the Strengths and Difficulties Questionnaire (SDQ; Goodman 1997; e.g., ‘unhelpful if someone is hurt, upset, or ill’, ‘not kind to younger children’). This combination of ‘callous’ and ‘low prosocial’ items has since been utilized extensively in youth studies of psychopathic traits (e.g., Dadds et al. 2006; Fontaine et al. 2011; Hawes et al. 2014; Viding et al. 2005). A recent attempt to clarify the relative shared and unique variance underlying IC and ‘low prosocial behavior’ (LPB) items within an epidemiological cohort found that a bifactor model provided the best fit for the two constructs (Meehan et al. 2019). Here, a general factor (termed ‘IC/LPB’) was associated with the highest levels of childhood risk exposure and psychiatric comorbidity. Additionally, based on recommended fit indices for bifactor models (see Rodriguez et al. 2016a, b), the residual factor for IC items did not appear to capture meaningful distinct variance over and above this general factor. However, an equivalent residual factor for LPB items, which here was associated with lower levels of parental warmth and higher levels of social-cognitive impairment, appeared to reflect some degree of unique variance. The emergence of a reliable ‘callous-low prosocial’ factor from these analyses may indicate conceptual overlap between the two constructs, such that a self-centered lack of consideration or concern for others may be a key feature of a callous interpersonal style.

While the combination of IC and LPB appears to index a more severe profile of early risk, little is known about how their co-occurrence may influence adjustment into adulthood. As outlined above, most research on youth callousness has focused on delinquency, with scant examination of wider health or functional outcomes. On the other hand, low prosocial behavior, as well as being associated with externalizing behaviors (Flynn et al. 2015; Nantel-Vivier et al. 2014), has also been linked to poor socio-emotional adjustment, including academic underachievement (Caprara et al. 2000; Gerbino et al. 2017). Of note, persistently high levels of CP predict greater mental and physical health difficulties in adulthood, along with worse education and employment outcomes (Fergusson et al. 2005; Moffitt et al. 2002; Odgers et al. 2007). In particular, young adults ‘not in education, employment, or training’ (NEET), a classification associated with chronic un-employment and poor physical and mental health (Franzén and Kassman 2005), are more likely to report histories of delinquency, substance abuse, and mental health problems (Rodwell et al. 2017; Veldman et al. 2015). Given that IC and LPB both designate early-onset and persistent CP, their shared variance (i.e., IC/LPB) may, in turn, be associated with adverse adult health and adjustment, whether directly or by initiating indirect developmental pathways via delinquency or related ‘health-compromising’ behaviors (e.g., substance use), which themselves increase the risk for a variety of negative health and behavioral outcomes (van Nieuwenhuijzen et al. 2009; Weinberg et al. 1998). At the same time, certain behaviors may confer promotive or protective effects against adverse early-adult outcomes. For example, physical activity is consistently associated with better health, emotional well-being, and academic achievement (Ahn and Fedewa 2011; Reiner et al. 2013; Spruit et al. 2016), while inactivity has been linked with delinquency (Morris and Johnson 2014).

It is also possible that any developmental pathways identified during adolescence may not be the result of IC/LPB per se, but instead reflect wider developmental vulnerability due to adverse early-life social contexts. Early-life adversity is prospectively associated with IC and LPB (Barker et al. 2011; Flouri and Sarmadi 2016), as well as delinquency, substance use, psychopathology, physical inactivity, and adverse health and socio-economic outcomes (Currie and Widom 2010; Dube et al. 2003; Felitti et al. 1998; Raposa et al. 2014; Taylor et al. 2011). In addition, cognitive deficits (i.e., low IQ) may also account for later health and adjustment problems: low IQ is predictive of an increased risk of unemployment at age 21 years (Caspi et al. 1998), while higher adolescent IQ has been linked with better health at age 40 based on self-reports and medical diagnoses (Der et al. 2009). Therefore, any proposed developmental models should adequately control for these potential confounding factors.

Consequently, the current study sought to extend a previous bifactor model (Meehan et al. 2019) to test prospective associations between these latent factors and early-adult measures of emotional well-being, physical health, and NEET status. Specifically, using three latent path models spanning from early adolescence to emerging adulthood, we aimed to examine direct effects between the relative shared and unique variances underlying IC and LPB and later health and social outcomes, over and above the effects of prior childhood adversity and intelligence, and test whether these constructs were indirectly related to early-adult outcomes via adolescent delinquency, substance use, and/or physical activity.

## Method

### Participants

Data were drawn from the Avon Longitudinal Study of Parents and Children (ALSPAC), a population-representative British birth cohort established to understand how genetic and environmental characteristics influence health and development in parents and children (Boyd et al. 2013; Fraser et al. 2013). Pregnant women resident in the former Avon Health Authority with expected delivery dates between 1 April 1991 and 31 December 1992 were eligible for recruitment, resulting in a cohort of 14,541 pregnancies, with 13,988 singletons/twins alive at 12 months. Ethical approval was obtained from the ALSPAC Ethics and Law Committee, as well as various Local Research Ethics Committees. Please note that the study website contains details of all the data that is available, through a fully searchable data dictionary: http://www.bris.ac.uk/alspac/researchers/data-access/data-dictionary/.

Participants with complete data at ages 18 or 20 ranged from 2,534–3,077, depending on the outcome measure. Within the smallest of these analytic samples, 62.9% of participants were female, compared to 49.7% at initial enrolment. It should be noted that this sample was 96.6% White; however, this is consistent with initial enrolment (96.1%; Boyd et al. 2013). In terms of socio-economic status (SES), 6.3% of mothers were classified as ‘low SES’, based on classes IV and V of the UK Registrar General’s social class scale (Office of Population Censuses and Surveys 1991), compared to 12% of the initial sample. To formally examine the impact of attrition, we used multivariate logistic regression with odds ratios (ORs) to test whether being male (OR = 2.12, 95% CIs = 1.92–2.34), low SES (OR = 1.54, 95% CIs = 1.28–1.84), early parenthood (<19 years; OR = 2.02, 95% CIs = 1.70–2.40), or low maternal educational attainment (basic school-leaving/vocational qualifications only; OR = 2.11, 95% CIs = 1.84–2.43) predicted exclusion from our smallest analytic sample (*n* = 2,534). All four variables were significantly associated with exclusion. However, previous analyses of bias in ALSPAC highlight that, although attrition impacted the prevalence of psychiatric disorders, associations between risks and outcomes generally remain intact, such that observed associations are likely to be conservative of true population effects (Wolke et al. 2009).

### Measures

#### Interpersonal Callousness

A six-item measure was completed by mothers when their child was 13 years old (Moran et al. 2008). Using a five-point scale (0 = ‘not at all’ to 4 = ‘always’), items rated the frequency that the child: (i) ‘makes a good impression at first, which people tend to see through after getting know him/her’; (ii) ‘has shallow or fast-changing emotions’; (iii) ‘is usually genuinely sorry if s/he has hurt someone or acted badly’ (reversed); (iv) ‘can seem cold-blooded or callous’; (v) ‘keeps promises’ (reversed); and (vi) ‘is genuine in his/her expression of emotions’ (reversed). Initial item selection was informed by factor analyses of scales measuring Factor 1 of the PCL–R (i.e., interpersonal-affective characteristics), the international standard for the assessment of psychopathy that has played a dominant role in the establishment of childhood measures (Frick et al. 2000; Frick et al. 1994). Validating these items among 182 clinic-referred or school-recruited children scoring highly for externalizing disorders, Moran et al. (2009) found a high correlation (r = .81) with the APSD’s (Frick and Hare 2001) Callous-Unemotional subscale. Internal consistency was acceptable within the current sample (α = .75).

#### Low Prosocial Behavior

The prosocial subscale of the Strengths and Difficulties Questionnaire (SDQ; Goodman 1997), a widely-used screening instrument with established reliability and validity, was completed by mothers when the child was aged 13. Five items assessed behavior ‘in the past six months’ along a three-point scale (0 = ‘not true’ to 2 = ‘certainly true’): (i) ‘considerate of other’s feelings’; (ii) ‘shares readily with other children’; (iii) ‘helpful if someone is hurt, upset, or ill’; (iv) ‘kind to younger children’; and (v) ‘volunteers to help others’. Responses were reversed, such that higher scores captured lower prosociality. Internal consistency was acceptable (α = .71).

#### Delinquency

Adolescent delinquency was measured using self-report items from the Edinburgh Study of Youth Transitions in Crime (Smith and McVie 2003) at ages 13 (15 items), 15 (16 items), 17 (17 items) and 18 years (12 items). Original response scales were dichotomized (0 = ‘no’; 1 = ‘yes’) based on whether respondents had partaken in a given activity in the past year (e.g., stole something from a shop, damaged property, broke into a house or building, been rowdy or rude in public place; see Table S1 for complete items at each time-point). Items were summed to create a total count of delinquent behaviors. Internal consistency was acceptable at all ages (α = .66–.82).

#### Substance Use

Self-reported tobacco and cannabis use was measured at ages 14, 16, and 18 years, using frequency items (0 = ‘never’ to 6 = ‘daily’). Alcohol consumption and alcoholrelated behaviors (also self-reported) were assessed at ages 16 and 18 years via the Alcohol Use Disorders Identification Test (AUDIT; Saunders et al. 1993), a 10-item screening tool developed by the World Health Organization. AUDIT items showed good internal reliability at ages 16 (α = .77) and 18 years (α = .75).

#### Physical Activity

Self-reported physical activity was assessed at ages 13, 16, and 18 years by asking ‘During the past year, how often did you do any exercise (going to the gym, brisk walking, or sports activity)?’ (range: 0 = ‘never’ to 4 = ‘5 or more times a week’).

#### Emotional and Physical Health Problems

Health outcomes at age 18 years were drawn from the 36-item Short Form Health Survey (SF-36; Ware and Sherbourne 1992). Emotional problems were represented by a latent factor derived from Mental Health (5 items; α = .80) and Role Emotional (3 items; α = .92) subscales. These captured the frequency of emotional problems in the past month (e.g., ‘been very nervous’, ‘felt downhearted or low’), and the burden of these problems on respondents’ everyday activities, respectively (e.g., ‘cut down on time spent on work or other activities’, ‘accomplished less than they would like’). Items were measured on a five-point scale (0 = ‘none of the time’ to 4 = ‘all of the time’).

A latent factor for physical health problems was derived from two subscales. First, nine items from the Physical Functioning subscale (α = .92) assessed the degree to which the respondent was physically limited in completing a range of activities (e.g., ‘lifting or carrying groceries’, ‘climbing several flights of stairs’, ‘walking one hundred meters’ etc.), rated from 0 (‘not limited’) to 2 (‘limited a lot’). Second, the Role Physical subscale (4 items; α = .92) captured the impact of respondents’ physical limitations on everyday activity in the past month (e.g., ‘limited in work or other activities’, ‘had difficulty performing work or other activities [i.e., involved extra effort]’), rated from 0 (‘none of the time’) to 4 (‘all the time’).

#### ‘Not in Education, Employment, or Training’ (NEET)

At age 20, as part of a wider questionnaire on education and employment, respondents were asked whether they were ‘currently in employment or doing any education or training’. The dichotomous response set was used to compare young people ‘not in education, employment, or training’ (NEET; coded 1) to the remainder of the sample (coded 0). This measure there-fore identifies young people not at school or work, for whatever reason.

#### Early-Life Adversity

Indicators of family, parental, and sociodemographic risk were collated under the short form of the Family Adversity Index (FAI; Bowen et al. 2005). This captured 15 family-based risk factors, including maternal age, educational qualifications, housing adequacy, financial difficulties, and maternal psychopathology, sub-stance abuse, and crime. An item was rated 1 if adversity was present, with scores summed to create a scale. Totals for two postnatal periods (birth to age 2, and age 2–4 years) were combined to create a cumulative early-life adversity score (range 0–20).

#### Intelligence

A short form of the Wechsler Intelligence Scale for Children, Third Edition (WISC-III; Wechsler et al. 1992) was administered at age 8 years. This comprises ten subtests of verbal and performance intelligence. The age-adjusted full-scale IQ score, representing the sum of all subtests, was utilized here.

### Statistical Analyses

We estimated separate latent path models for each outcome, using Mplus v7.11 (Muthén and Muthén 2012): (i) emotional problems (Model A); (ii) physical health problems (Model B); and (iii) NEET status (Model C). For each model, only those with complete data for the outcome were included in analysis, with all models controlling for childhood adversity and IQ. With the exception of the binary NEET outcome, latent factors were estimated for each intermediary (adolescent delinquency, substance use, physical activity) and outcome domain (emotional problems, physical health problems) to maximize shared variance between indicators and minimize inclusion of error variance (Skrondal and Rabe-Hesketh 2004). The underlying latent factor structures for each model are presented in Figs. S1–S3.

At age 13, we replicated (Meehan et al.’s 2019) bifactor model, specifying a general factor (IC/LPB) for shared variance among all items and specific IC and LPB factors for residual covariance among item subsets, with all covariance between factors fixed to zero (Brown 2006). Recent work has raised concerns around the propensity of bifactor models to unwittingly capture measurement error or ‘noise’ variance, rather than theoretically-distinct constructs, such that associations with external variables were likely to be unreliable (Bonifay et al. 2017; Rodriguez et al. 2016b). To address this issue, we derived a series of bifactor-specific fit indices using the Omega program (Watkins 2013) to assess the reliability and construct validity of estimated factors. These indices and their interpretation are summarized below; for comprehensive accounts of their calculation, see Rodriguez et al. (2016a, b).

First, coefficients omega hierarchical (*ω*_*H*_) and hierarchical subscale (*ω*_*HS*_) were used as model-based reliability estimates, analogous to coefficient alpha. Specifically, *ω*_*H*_ measures the proportion of systematic variance in the unit-weighted total score attributable to individual differences on the general factor, while *ω*_*HS*_ assesses the proportion of subscale score variance accounted for by individual differences on its intended specific factor after controlling for variance explained by the general factor. Although *ω*_*H*_ or *ω*_*HS*_ values closer to >.75 are preferred, there are no absolute standards, and values >.50 can indicate sufficient reliability (Reise et al. 2013). Second, construct reliability, or the extent to which a latent factor is represented by its underlying items, was measured using *H*, where a value of .70 indicates adequate representation, such that the factor is likely to replicate well across samples. Finally, explained common variance (ECV) and percentage of uncontaminated correlations (PUC) were used to assess the relative multidimensionality of the bifactor model. ECV is the proportion of variance explained by all factors that is accounted for by the general factor, while PUC indicates the proportion of correlations between items that are influenced by the general factor. Where both ECVand PUC are >.70, the data may be thought of as essentially unidimensional, to the extent that estimating a single latent factor may be more parsimonious than the bifactor structure (Rodriguez et al. 2016a).

For Models A and B, maximum likelihood estimation with robust standard errors (MLR) was used to correct for possible non-normal distribution of study variables. As the NEET outcome was binary, robust weighted least squares (WLSMV) estimation was used for Model C. In all models, full information maximum likelihood (FIML) was used to account for missing data. Model fit was evaluated using the comparative fit index (CFI) and Tucker-Lewis index (TLI), where values ≥.90 and ≥ .95 represent acceptable and good fit, respectively (Hu and Bentler 1999), and the root mean square error of approximation (RMSEA), where values ≤.08 and ≤ .05 suggest adequate and close fit, respectively (Browne and Cudeck 1993). These thresholds were originally derived for continuous data (i.e., Models A and B). However, evaluating fit indices in models with binary outcomes using WLSMV (i.e., Model C), Yu (2002) found these cut-off values to be broadly applicable where *N* ≥ 250.

We inspected developmental pathways in two steps:

- **Step 1: Direct effects.** For each model, we estimated the following direct effects: (i) IC/LPB, IC, and LPB → earlyadult outcome (emotional problems, physical health problems, or NEET status); (ii) IC/LPB, IC, and LPB → delinquency, substance use, and physical activity; and (iii) delinquency, substance use, and physical activity → earlyadult outcome.
- **Step 2: Mediation and indirect effects.** Indirect effects were tested using the ‘model indirect’ Mplus command and bootstrapped 10,000 times with bias-corrected 95% confidence intervals to account for non-normality in standard errors. We distinguish between mediation and indirect effects here. Mediation is contingent on a significant direct effect between predictor and outcome (or *c*-path) being explained by an intervening variable (Preacher et al. 2007). This is represented as the product of the pathways from predictor → mediator (*a*-path) and mediator → outcome (*b*-path), or the *ab* pathway. In contrast, indirect effects, though calculated in the same way, do not require a significant direct effect (i.e., *c*-path) to be present (Mathieu and Taylor 2006). Effect sizes for indirect pathways were represented by *P*_*M*_, or the ratio of indirect effect to total effect, as recommended by Wen and Fan (2015) for multiple-mediation models with large samples (*N* > 500).

## Results

**Fig. 1:**
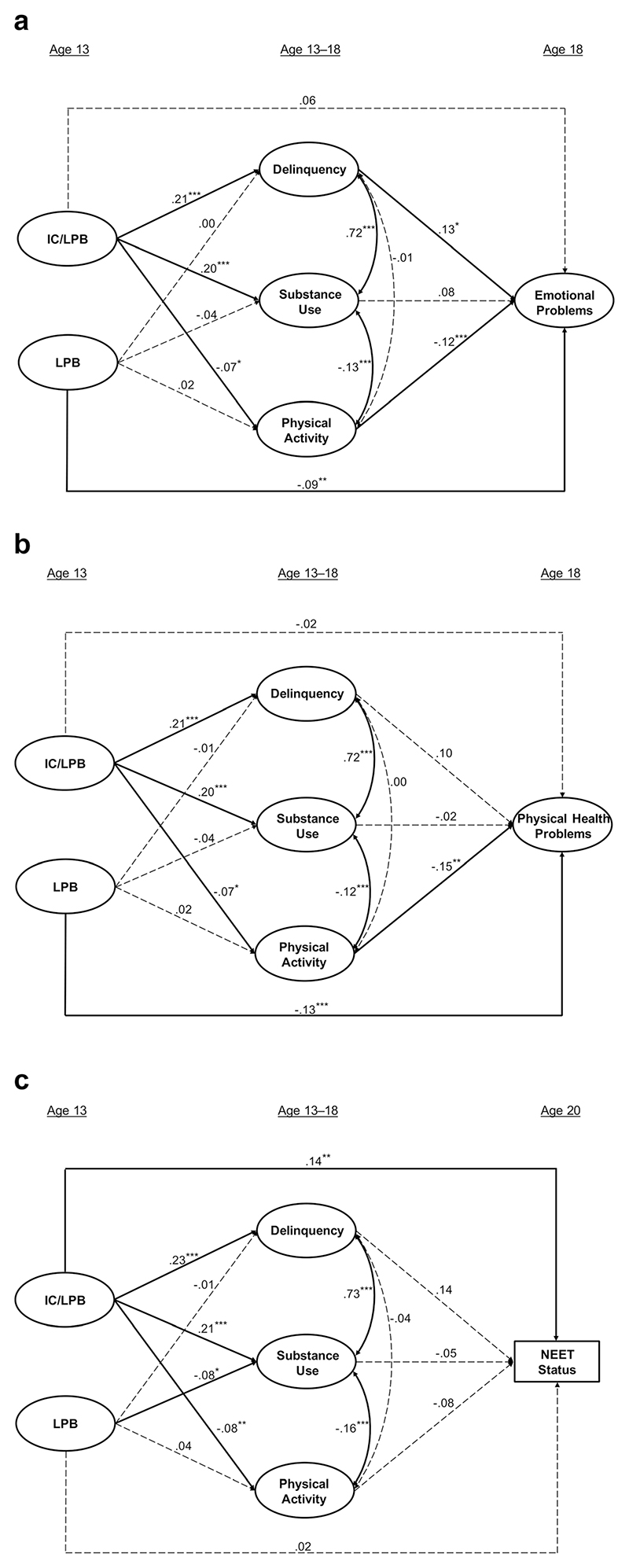
Standardized path estimates for predictors of (**a**) emotional problems (*n* = 2,541); (**b**) physical health problems (*n* = 2,534); and (**c**) NEET status (*n* = 3,077). All associations control for early adversity and childhood intelligence. Observed indicators for latent factors (circles) are not shown (see Fig. S1–S3 for underlying factor structures). Broken lines indicate nonsignificant associations (i.e., *p* > .05). *p < .05, **p < .01, ***p < .001

First, before proceeding with item-level analysis, the correlation (*r* = .49) between manifest (i.e., summed) IC and LPB scores was noted, suggesting that they represented moderately related, but nonetheless distinct, domains. Standardized pathways for the three path models, hereafter referred to as Model A (emotional problems; *n* = 2,541), Model B (physical health problems; *n* = 2,534), and Model C (NEET status; *n* = 3,077), are presented in Fig. 1a–c. All three models fit the data adequately (see Table 1), and latent structures were consistent across all three models (for item loadings among latent factors, see Figs. S1–S3). Bivariate correlations between latent factors within each model can be found in Tables S2–S4.

Additional fit indices for each of the three bifactor measurement models (see Table 2) revealed that, similar to previous findings in this cohort (Meehan et al. 2019), the specific IC factor (i.e., residual variance for IC items having accounted for variance shared with LPB) showed relatively poor reliability. *ω*_*HS*_ statistics across the three models suggested that this factor only accounted for 4.9–11.5% of the variance in the proposed IC subscale score, having partitioned out variance explained by the general factor. In addition, the latent factor appeared to be a poor representation of the IC items themselves (*H* = .25–.28). Given its apparent unreliability, we did not specify longitudinal associations between the latent IC factor and adolescent or early-adult latent factors within the three models.

Elsewhere, high *ω*_*H*_ values for the general factor (i.e., IC/LPB) indicated that the majority (68.4–71.6%) of total score variance was accounted for by individual differences on this latent factor, while *H* values (.81–.83) indicated a factor that was well-defined by the 11 items. At the same time, across the three models, there was no instance where both ECV and PUC values exceeded .70. This suggested that although the IC/LPB factor explained the majority of variance among these items, a non-trivial amount of variance was explained by the specific factors (Rodriguez et al. 2016a). Of note here, although neither *ω*_*HS*_ (.35–.38) or *H* values (.52–.53) for the residual LPB factor reached accepted thresholds, this latent factor showed better overall reliability in all three models compared to the residual IC factor. Therefore, in an effort to capture some of the multidimensionality suggested by the sub-optimal combination of ECV and PUC values, we specified associations between this factor and other latent domains in each model. These associations are described in subsequent sections; however, given its poorer reliability, we acknowledge that our examination of associations involving this LPB factor was more exploratory in nature than those involving the highly-reliable IC/LPB factor, which were more likely to be robust to replication.

Direct and indirect pathways are now discussed for each model:

- **Step 1: Direct effects**
**Model A: Emotional Problems.** As shown in Fig. 1a, IC/LPB at age 13 was not directly associated with emotional problems at age 18; however, higher LPB was associated with *lower* levels of emotional difficulties (*b* = –.09, *p* = .006). In terms of intermediate direct effects, IC/LPB was associated with higher levels of delinquency (*b* = .21, *p* < .001) and substance use (*b* = .20, *p* < .001), and lower levels of physical activity (*b* = –.07, *p* = .023). The residual LPB factor did not significantly associate with any of the adolescent domains. Greater delinquency (*b* = .13, *p* = .024) and lower physical activity (*b* = –.12, *p* < .001) were, in turn, directly associated with greater emotional difficulties.
**Model B: Physical Health Problems.** Similar to Model A, although IC/LPB was not directly associated with physical health problems, higher LPB was directly associated with *better* physical health (*b* = –.13, *p* < .001; see Fig. 1b). However, associations between IC/LPB and LPB and adolescent domains (delinquency, substance use, and physical activity) produced the same pattern of effects, with broadly equivalent standardized estimates, as reported in Model A above. Finally, with regard to these adolescent behaviors, lower levels of physical activity were related to higher levels of physical health difficulties (*b*= –.15, *p* = .001). Neither delinquency nor substance use significantly predicted physical health.
**Model C: NEET Status.** Youth defined as NEET (*n* = 212; 6.9%) were coded 1, with the remainder of the sample (*n* = 2,865; 93.1%) coded 0. Therefore, a positive association suggested that higher levels of a given factor were associated with NEET classification at age 20. As seen in Fig. 1c, higher IC/LPB at age 13 was directly associated with NEETstatus (*b*= .14, *p* = .001). No direct effect was observed for the LPB factor. Associations between IC/LPB and the adolescent variables were broadly similar to the previous two models, with one additional significant association: here, higher LPB was significantly related to lower substance (*b*= –.08; *p* = .014). Finally, none of the three adolescent domains were significantly associated with NEET status.
- **Step 2: Mediation and indirect effects**
  Significant indirect pathways are presented in Table 3. As no significant mediation was found, these pathways represent indirect effects (i.e., where the direct effect is non-significant). None of the bootstrapped 95% confidence intervals for these effects crossed zero. Full results of indirect effects analysis, including total effects, are available in Table S5.
**Model A: Emotional Problems.** One significant indirect effect was found: IC/LPB was associated with greater emotional problems via higher levels of adolescent delinquency (*b* = .028, *SE* = .013, *p* = .034, *P*_*M*_ = .26).
**Model B: Physical Health Problems.** One indirect effect was observed in this model: IC/LPB was associated with greater physical health problems, via lower levels of physical activity (*b* = .017, *SE* = .007, *p* = .009, *P*_*M*_ = 1.13). As direct and indirect effects had opposite signs, the estimate for the indirect effect was greater than that of the direct effect, resulting in a *P*_*M*_ value >1. To provide an alternative measure of the size of this effect, this pathway accounted for 43.6% of the total indirect effect from IC/LPB to physical health issues.
**Model C: NEET Status.** No significant indirect pathways were identified here.

## Discussion

This study sought to evaluate the predictive utility of the shared and unique variances for IC and LPB on early-adult health and educational/occupational attainment, via direct and indirect developmental pathways. Four main findings are highlighted from these analyses.

First, having controlled for childhood adversity and cognitive function, the shared variance underlying these 11 items (i.e., IC/LPB), which accounted for the majority of total score variance, was indirectly associated with young-adult emotional difficulties via higher levels of adolescent delinquency. Longitudinal research has demonstrated the utility of adolescent callousness in predicting more delinquent and criminal outcomes in adulthood (e.g., McMahon et al. 2010; Kahn et al. 2013). Meanwhile, persistently high levels of youth conduct problem behaviors have themselves been shown to predict poorer adult mental health (e.g., Fergusson et al. 2005; Odgers et al. 2007). Therefore, these findings propose a developmental pathway through which these three domains may inter-relate over time; specifically, the severe and persistent pattern of antisocial behavior that characterizes high-callous youth (Frick et al. 2014) can be an adverse outcome in itself, and indirectly give rise to wider self-reported mental health difficulties in young adulthood.

Second, lower physical activity engendered an indirect effect between IC/LPB and physical health problems. Physical activity has established benefits on long-term health and well-being (Penedo and Dahn 2005; Reiner et al. 2013), and confers psychosocial resources that contribute to overall life success, most notably social support (Hogan et al. 2015; Mendonça et al. 2014). Participation in physical activity is strongly influenced by parental or peer support (Beets et al. 2010). At the same time, organized group activities (e.g., team sports) can themselves increase social support by providing more opportunities for social connectedness compared to individual healthrelated behaviors (Eime et al. 2013). Therefore, lower levels of physical activity at high levels of IC/LPB may also reflect poor social support. However, not all group activities are associated with positive adjustment: unstructured and unsupervised activities (e.g., hanging out with peers) generally increase the risk of delinquent or risky behaviors, compared to the protective effects conferred by participation in structured, organized, and adult-supervised activities (e.g., Hoeben and Weerman 2016; Osgood et al. 1996). Given that our measure simply assessed the average weekly frequency of any exercise, it was not possible to distinguish activity subtypes (i.e., independent vs teambased, structured vs unstructured), prohibiting formal examination of any additional mechanisms.

Third, IC/LPB was directly associated with nonparticipation in education, employment, or training at age 20. The proportion of NEET youth in our sample (6.9%) was lower than the 14.9% rate reported for UK youth aged 16–24 at the time (April–June 2013; Office of National Statistics 2013). Of note, although IC/LPB was associated with greater delinquency and substance use, these behaviors were not predictive of NEET status, unlike previous longitudinal findings (Rodwell et al. 2017; Veldman et al. 2015). We suggest two reasons why our results differed from these studies. First, given that these adolescent variables were independently correlated with NEET status (see Table S4), it could be that prospective effects were accounted for by our controls here. Our adversity index included indicators of maternal education attainment and marital status, and we also controlled for IQ; all of these are robust independent risk factors for becoming NEET. In a similar way, Fergusson et al. (2005) found that an association between childhood CP and adverse adult psychosocial outcomes became non-significant once confounding factors, including family socio-economic disadvantage and IQ, were controlled for. Second, alternative developmental pathways not measured here may be more appropriate for poor academic or occupational success. For example, high-IC youth have been shown to perform worse on national standardized tests (Meehan et al. 2017). Academic failure, an established NEET risk factor, could therefore offer an indirect pathway from IC/LPB to NEET status by restricting later opportunities. In addition, it may be more pertinent to examine school-based behaviors (e.g., truancy, deviant peer affiliation, exclusion, teacher-child conflict), as school engagement and academic achievement are highly inter-related (Chase et al. 2014). As well as exerting unique effects, indicators of school disengagement may engender an indirect effect between delinquency or substance use and NEET status, as a more proximal measure of how these behaviors relate to later academic failure.

Finally, the specific LPB factor, or unique variance for low prosocial behavior beyond IC/LPB, was directly associated with *fewer* mental and physical health problems, suggesting a relatively well-adjusted developmental profile compared to the general factor. This unique variance for low prosocial behavior has previously been characterized by lower maternal warmth (Meehan et al. 2019). In line with consistent links between parental warmth and prosocial behaviors (Eisenberg et al. 2015), these youth may have less positive socialization, and hence be less considerate, due to the types of parenting behaviors to which they were exposed. Perhaps poor prosocial functioning limits interactions with peers, which, in turn, lowers the risk of affiliation with delinquent peers – delinquency, by and large, is believed to be (in part) a social behavior (Warr 2002). Consequently, they may not become involved in activities that compromise later health (e.g., delinquency, substance use). Elsewhere, while this LPB factor was previously related to greater social-cognitive deficits, these may reflect low-level difficulties within a normative community sample, rather than clinically-relevant impairments (i.e., autism diagnosis). These are speculative suggestions, however, as observed associations should be interpreted with caution given the factor’s suboptimal bifactor fit indices. Moreover, as this LPB factor represents *residualized* variance, having partitioned out shared variance with IC, the extent to which the factor adequately reflects the original LPB construct is unclear, limiting meaningful interpretation of these effects. Incorporating further domains of functioning (e.g., bullying, friendship quality) may address uncertainty around the reliability of the LPB factor by offering a more nuanced picture of the behavioral profile associated with this residualized variance.

### Clinical Implications

Overall, findings suggest that IC/LPB, which may represent a similar construct as previous empirically derived ‘callous-low prosocial’ measures (Dadds et al. 2005; Viding et al. 2005), is also predictive of adverse health and social adjustment, in what is, to our knowledge, the first prospective study of these outcomes in relation to IC and LPB. By identifying indirect developmental pathways for IC/LPB, these results, in turn, highlight several targets for early clinical intervention that may mitigate the potential for long-term disadvantage among IC/LPB youth, even where levels of initial risk are high, as denoted by a more adverse early social context and poorer cognitive function.

First, efforts to reduce delinquency in adolescence may also promote better emotional well-being. High-callous youth, though not entirely unresponsive, have previously benefitted less from standard parent-training interventions aimed at reducing conduct problems compared to youth without these traits (Hawes et al. 2014). However, recent treatment efforts have devised more precise treatment targets for this subgroup of CP youth. Of note, greater reductions in CP have been reported for callous youth following interventions that include an emotion recognition training component (Dadds et al. 2012). School-based prevention training focused on increasing emotional awareness and social skills has also reported preliminary success in reducing symptoms of CP and callousness (Kyranides et al. 2017). Treatment designs that address these socio-affective skills may therefore reduce delinquent behaviors and, in turn, lessen overall risk for emotional difficulties into early adulthood. Adolescent levels of delinquency observed here are also likely to reflect continuity of these behaviors from childhood, as CP youth with elevated IC levels tend to show an earlier onset and more stable pattern of antisocial behavior (Frick et al. 2014). Consequently, preventive approaches are needed in early childhood, before the onset of severe problems, and when IC may be less likely to moderate intervention effects on problem behaviors (Hyde et al. 2013).

Second, promoting physical activity, or reducing sedentary and inactive behaviors, may improve long-term physical health, even among IC/LPB youth, who, based on these results, are more likely engage in risky or ‘health-compromising’ behaviors. Exercise-based interventions among school-aged children generally show a dose-response relationship with physical health: the more activity, the greater the health benefit (Janssen and LeBlanc 2010). Such interventions have also proven effective as adjuncts to mental health treatments (Josefsson et al. 2014; Stathopoulou et al. 2006; Rosenbaum et al. 2016). Despite evidence for a beneficial effect of these interventions on mental as well as physical health, no published study to date has examined exercise within treatment for callous traits. Given that youth high in callousness, irrespective of CP levels, report lower levels of self-esteem, social support and peer functioning (Fanti 2013; Haas et al. 2017), promoting group activity such as team sports may be particularly efficacious, due to the additional opportunities to increase social connectedness and improve social interaction skills.

### Strengths and Limitations

The current study benefitted from a developmentally-informed design, large sample sizes, and availability of repeated measures from multiple informants. Nonetheless, several limitations must be acknowledged. First, although data are longitudinal, conclusive casual effects cannot be established, as alternative models may offer equally plausible explanations for the data (Masten and Cicchetti 2010). In particular, unmeasured ‘third-cause variables’ may account for some effects; for example, shared genetic predispositions may underpin several of the measured domains, including IC and LPB themselves. Consequently, these models represent one of many possible configurations, and future work should assess the robustness of these effects by evaluating alternative solutions. Second, selection of time-points (e.g., IC) was based on the availability of data, rather than specific hypotheses about sensitive developmental periods. This limits conclusions that can be drawn regarding the role of timing, as effects and behaviors may have emerged earlier in development than their point of first measurement in ALSPAC. Repeated measures may help to specify critical periods of developmental vulnerability. Third, these models adopt a variable-based approach within a normative sample, comparing relative scores on continuous dimensions rather than categorical subgroups (e.g., classifying ‘high-IC’ or ‘high-LPB’ youth). It is unclear how findings might differ in high-risk samples, or at clinical cut-offs in the underlying distributions for IC or LPB. Fourth, our non-specific measure of physical activity did not differentiate between independent and group activities. More nuanced measures could clarify whether specific activities offer a greater promotive effect against adverse physical health. Fifth, although ALSPAC features a broad spectrum of socioeconomic backgrounds, the cohort includes relatively low rates of ethnic minorities, necessitating replication in more diverse samples. Finally, as discussed previously, attrition in ALSPAC has led to the loss of more vulnerable families at follow-up. However, Wolke et al. (2009) concluded that, although attrition affected overall prevalence rates for psychiatric disorder in ALSPAC, it did not attenuate relationships between risks and outcomes, provided that attrition bias was based around the risk factor rather than the outcome.

## Conclusions

In summary, this study showed that, controlling for high childhood social disadvantage and low intelligence, the shared variance underlying IC and LPB in early adolescence was directly associated with young-adult non-participation in education and employment, and indirectly associated with adverse mental and physical health in young adulthood via delinquent and physically-active behaviors, respectively. Residual variance for LPB, in contrast, was related to better mental and physical health. These longitudinal models offer preliminary evidence for a number of developmental pathways through which IC/LPB may exert enduring effects on general health and adjustment, and highlight two appropriate targets for multicomponent treatment efforts during adolescence, focused on both the reduction of delinquent or disruptive behaviors and promotion of physical activity.

## Supplementary Material

**Electronic supplementary material** The online version of this article (https://doi.org/10.1007/s10862-019-09756-9) contains supplementary material, which is available to authorized users.

Supplementary material

## Figures and Tables

**Table 1:** Model fit information for estimated latent path models

	Model fit statistic				
	χ^2^ (*df*)	*p* value	CFI	TLI	RMSEA (90% CIs)
Model A (emotional problems)	2,539.64 (559)	<.001	.92	.91	.037 (.036–.039)
IModel B (physical health problems)	3,520.67 (744)	<.001	.90	.90	.038 (.037–.040)
Model C (NEET status)	2,137.90 (345)	<.001	.91	.90	.041 (.039–.043)

*NEET* not in education, employment, or training, χ^2^ chi-square statistic, *CFI* comparative fit Index, *TLI* Tucker-Lewis index (acceptable fit for both: ≥.90), RMSEA root mean square error of approximation (close fit: ≤.05), CIs confidence intervals

**Table 2:** Bifactor-specific fit indices for general and specific factors at age 13 within each latent path model

Latent factor	Bifactor-derived statistic			
	*ω*_*H*_ / *ω*_*HS*_	*H*	ECV	PUC
Model A (emotional problems)				
IC/LPB	.716	.824	.708	.545
IC	.049	.262		
LPB	.377	.535		
Model B (physical health problems)				
IC/LPB	.715	.825	.709	.545
IC	.052	.253		
LPB	.377	.535		
Model C (NEET status)				
IC/LPB	.689	.809	.696	.545
IC	.115	.283		
LPB	.352	.518		

*ω*_*H*_ omega hierarchical (for IC/LPB), *ω*_*HS*_ omega hierarchical subscale (for IC and LPB), *H* construct reliability, *ECV* explained common variance, *PUC* percentage uncontaminated correlations, *NEET* not in education, employment, or training

**Table 3:** Significant standardized indirect pathways for emotional problems and physical health problems

Age 13	Age 13–18	Age 18	Estimate	*SE*	*p* value	95% bias-corrected CIs		*P*_*M*_
						Lower	Upper	
Model A								
IC/LPB [+]	Delinquency [+]	Emotional Problems [+]	.028	.013	.034	.002	.054	.26
Model B								
IC/LPB [+]	Physical Activity [-]	Physical Health Problems [+]	.017	.007	.009	.004	.030	1.13^a^

[+] = increasing; [–] = decreasing; CIs = confidence intervals; *P*_*M*_ = ratio of indirect effect to total effect.

a *P*_*M*_ is >1 due to opposite signs for the direct and indirect effect; this specific indirect effect represented 43.6% of the total indirect effect from IC/LPB to physical health problems
